# Campylobacter outbreak associated with raw drinking milk, North West England, 2016

**DOI:** 10.1017/S0950268820000096

**Published:** 2020-01-31

**Authors:** J. Kenyon, T. Inns, H. Aird, C. Swift, J. Astbury, E. Forester, V. Decraene

**Affiliations:** 1 Public Health England North West Centre, Preston, UK; 2 Field Service, National Infection Service, Public Health England, London, UK; 3 Food, Water & Environmental Microbiology Laboratory, Public Health England, York, UK; 4 Gastrointestinal Bacterial Reference Unit, Public Health England, London, UK

**Keywords:** Campylobacter, epidemiology, food-borne infections, outbreak, raw milk

## Abstract

In December 2016, Public Health England investigated an outbreak of campylobacteriosis in North West England, with 69 cases in total. Epidemiological, microbiological and environmental investigations associated the illness with the consumption of unpasteurised cows' milk from Farm X, where milk was predominantly sold from a vending machine. Campylobacter was detected in milk samples which, when sequenced, were identical in sequence type as pathogens isolated from cases (Clonal Complex ST-403, Sequence Type 7432). The farm was served with a Hygiene Emergency Prohibition Order to prevent further cases. To our knowledge, this is the first outbreak of campylobacter associated with unpasteurised milk in England since 1996. Our findings highlighted several important lessons, including that the current testing regime in England for unpasteurised milk is not fit for purpose and that the required warning label should include additional wording, underscoring the risk to vulnerable groups. There has been a substantial increase in both the volume of unpasteurised milk consumed in England and the use of vending machines to sell unpasteurised milk over the last 10 years, making unpasteurised milk more readily accessible to a wider population. The evidence generated from outbreaks like this is therefore critical and should be used to influence policy development.

## Introduction

Campylobacter is the most frequently reported cause of food poisoning in the UK. In 2016, there were 90.2 confirmed cases per 100 000 population reported in the UK [1], a total of 58 987 confirmed laboratory campylobacter cases. The true incidence of campylobacter infection, however, is higher due to underreporting [2].

Raw, unpasteurised milk is a well-known cause of campylobacter outbreaks, with numerous reported outbreaks from the UK and elsewhere [2–9]. Contamination of raw cows drinking milk (RDM) mainly occurs during the milking process, most commonly via faecal contamination of udders [10, 11] while the failure of milk pasteurisation is a recognised cause of campylobacter outbreaks. Due to these risks, in the UK, RDM is controlled through legislation which prohibits or restricts its production and sale. Schedule 6 of the domestic Food Safety and Hygiene (England) Regulations 2013 restricts RDM sales in England, only permitting sales from an RDM producer directly to the final consumer, with equivalent law in Wales and Northern Ireland. Legislation in Scotland prohibited the sale of RDM in 1983 [12]; this was as a direct consequence of several fatal food poisoning outbreaks with links to RDM consumption [13].

Despite legislation there continue to be outbreaks of gastroenteritis linked to RDM consumption in England. Data from Public Health England (PHE) show that in England and Wales, between 1992 and 2002, there were 17 outbreaks of gastroenteritis reported that were linked to RDM, while none were reported between 2003 and 2013. Six gastroenteritis outbreaks linked to RDM were reported between 2014 and 2017; implicated pathogens included Shiga toxin-producing *Escherichia coli* O157, *Salmonella* Dublin and *Campylobacter* spp. [14].

Most Environmental Health Officers (EHOs) in North West England do not complete exposure questionnaires for campylobacter cases; therefore, surveillance of campylobacter relies on laboratory reporting. In some local authorities, such as Cumbria, campylobacter cases are sent postal questionnaires. In early December 2016, a local authority in Cumbria, North West England, received surveillance questionnaires from two cases of campylobacter who reported consuming RDM bought from Farm X. Upon identifying this link, EHOs pro-actively followed up a limited number of campylobacter cases and identified a further three cases who had consumed RDM from Farm X. The five cases had onset dates over a 12-day period and all reported consuming RDM on separate occasions from the same dairy farm (Farm X). Farm X started selling RDM in March 2016. This dairy farm had a café and farm shop in which RDM was served and RDM was also sold via an outdoor self-service vending machine. An outbreak was declared and we describe the subsequent public health investigations and management in order to highlight issues with routine RDM microbiological monitoring practices and labelling of RDM.

## Methods

### Epidemiological

A review of campylobacter cases in the area surrounding Farm X identified an increase in cases in November 2016; this therefore defined the start of the study period. A confirmed case was defined as a person who had onset of diarrhoea between 3 November and 31 December 2016, had visited Farm X up to 10 days before the onset of illness and had *Campylobacter* spp. isolated from a stool sample. A probable case was as above but without microbiological confirmation. Diarrhoea was defined as two or more loose stools within 24 h. Cases were excluded if they had a history of foreign travel or contact with a household member with diarrhoea and/or vomiting in the 10 days before illness onset. We also conducted active case finding: reported campylobacter cases in the surrounding area that had dates of onset of illness consistent with the case definition were contacted and asked specifically about raw milk consumption from Farm X.

A retrospective cohort study was undertaken to identify the source of illness. The cohort was defined as persons who had visited Farm X in the study period (3 November to 31 December 2016). Potential study participants were reached through a press statement issued on 22 December 2016 which was covered extensively by local media sources. A link to an online questionnaire was included in this press release, this questionnaire collected data until 4 January 2017. The online questionnaire asked for information including dates of illness onset, clinical symptoms, details about their consumption of RDM and other items at Farm X.

For the descriptive analysis, all cases were included: cases identified from the cohort study as well as those identified from active case finding activities.

Retrospective cohort data were analysed using Stata 12.0 (College Station, Texas, USA). Cases and non-cases were described and compared using *t* test for continuous variables and Fisher's exact test or *χ*^2^ test as appropriate for categorical data. The outcome was illness meeting the case definition and the main exposure was RDM. The association between illness and each variable (including demographic factors and foods consumed) was estimated using odds ratios (ORs); 95% confidence intervals around these estimates and *P*-values were also calculated. Univariable and multivariable analyses were conducted using logistic regression. Variables which had a *P* value <0.05 in univariable analysis were included in the multivariable model.

### Microbiological: human samples

Stool samples from outbreak cases were sent to local hospital laboratories for diagnostic testing. All viable human campylobacter isolates were further typed and sequenced at the Gastrointestinal Bacteria Reference Unit, PHE, Colindale, London using whole genome sequencing (WGS). Local hospital laboratories store campylobacter isolates for 3 months after isolation; after this point, they are discarded.

### Microbiological: environmental samples

Farm X was visited by Dairy Hygiene Inspectors (DHI) from the Food Standards Agency (FSA); the dairy, milking parlour and cattle housing areas were inspected on multiple occasions over the course of the outbreak investigation. Environmental samples were taken from a bore hole private water supply, the bulk milk tank and dairy equipment. No cattle samples were taken. EHOs from the local authority also visited Farm X to review the RDM vending machine and hygiene practices in the café and at the farm shop.

The PHE Food, Water and Environmental (FWE) Microbiology Laboratory in York carried out testing of RDM and environmental samples. The FWE laboratory methods used to detect campylobacter were based on BS EN ISO 10272-1:2006 [15]. This involves enrichment in a selective liquid medium at 37 °C for 5 h followed by microaerobic incubation at 41.5 °C for 44 h to allow recovery and growth, sub-culture onto selective solid media, and examination for colonies considered to be typical of campylobacter species. RDM samples from the farm premises were collected on 13 December 2016 (vending machine) and 22 December 2016 (RDM bulk tank) and analysed at the FWE laboratory. Confirmation of the colonies as *Campylobacter* spp. was performed using morphological, biochemical and growth property tests and PCR [16]. Positive RDM campylobacter isolates were then sent to the Gastrointestinal Bacteria Reference Unit for WGS analysis.

### WGS and analyses methods

DNA was extracted and purified from isolates of campylobacter using the QIAsymphony DSP DNA kit on a QIAsymphony SP automated DNA extraction platform (Qiagen, Manchester, UK) according to manufacturer instructions. Genomic DNA was subsequently sequenced by the PHE Genomics Development and Services Unit as described previously [17]. FASTQ reads were quality trimmed using Trimomatic [18] as previously described [19]. Sequence type (ST) assignment was performed using MOST [20] and assigned a clonal complex (CC) in accordance with the PubMLST scheme (https://pubmlst.org/campylobacter/). Isolates of ST-403 CC were mapped against reference genome SRR9852498 as previously described [19] with single nucleotide polymorphism (SNP) positions determined using SnapperDB [21]. FASTQ sequences from raw milk samples (SRR10006879; SRR10006844) and human samples (SRR10011442; SRR10002190; SRR10001475; SRR10002325; SRR10001313; SRR10002375; SRR10002306) were deposited in the National Center for Biotechnology Information Short Read Archive under the BioProject PRJNA505131.

## Results

### Epidemiological results

**Fig. 1.**
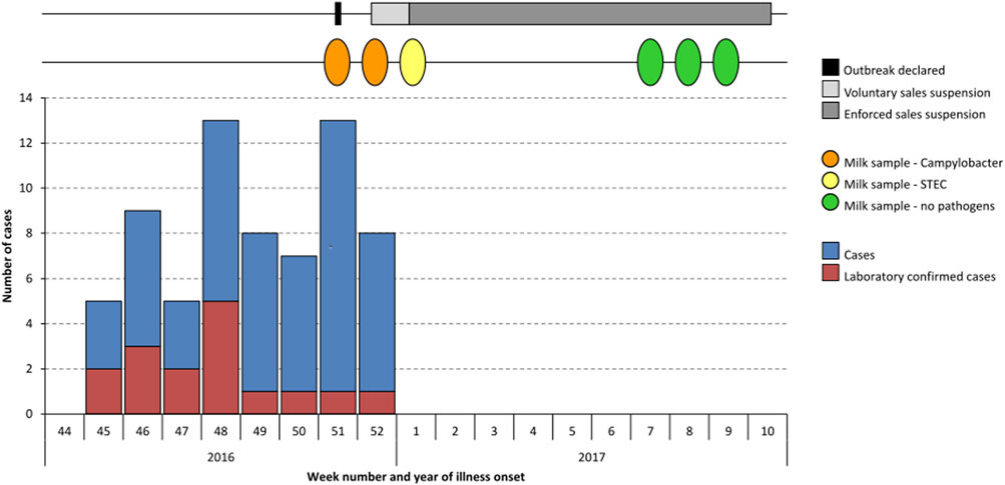
Epidemic curve showing dates of case illness onset, raw milk sampling and suspension of raw milk sales (*n* = 69).

In total, 69 cases were detected from active case finding activities and the retrospective cohort study. Duration of illness ranged from 1 to 32 days, with a median duration of 5 days. The mean age of cases was 44 years with a range from 1 to 74 years. Case onset dates of symptoms ranged from 3 November to 25 December 2016. Case onset dates are shown in Figure 1. No cases were hospitalised.

The retrospective cohort study included 292 participants; 63 cases and 229 non-cases. The mean age of non-cases was 52 years (range 5–86 years) whereas the mean age of cases was 44 years (range 1–74 years), a statistically significant difference (*P* = 0.0006). Fifty-three per cent of respondents were female. Cases were predominantly male, 61.9% compared to only 42.4% of non-cases; this difference was also statistically significant (*P* = 0.006).

Although the study undertaken was a retrospective cohort, due to the low response rate, we calculated ORs, rather than relative risks. In univariable analysis, we found that cases had significantly higher odds of having consumed raw milk than non-cases: OR 4.6 (95% confidence interval 1.87–13.67, *P* < 0.0001). Ninety-one per cent of cases (57/63) had consumed RDM. Cases were also twice as likely to be male: OR 2.2 (95% confidence interval 1.2–4.11, *P* = 0.006). Table 1 shows the univariable analysis results; age was assessed as a continuous variable and therefore is not presented in the table (OR 0.97, 95% confidence interval 0.95–0.99, *P* = 0.001).

**Table 1.** Results of the univariable analysis, reporting odds ratios (OR) with 95% confidence intervals (95% CI) for exposures for cases and non-cases, listed by significance level

Exposure	Cases			Non-cases			OR	95% CI	*P* value
	Exposed	Total	%	Exposed	Total	%			
RDM^a^	57	63	90.5	154	229	67.3	4.6	1.87–13.67	<0.0001
RDM from vending machine only	32	63	50.8	61	229	26.6	2.8	1.53–5.25	<0.0001
Tap water	2	63	3.2	39	229	17.0	0.2	0.02–0.65	0.005
Sex (male)	39	63	61.9	97	229	42.4	2.2	1.20–4.11	0.006
Other drink	8	63	12.7	66	229	28.8	0.4	0.14–0.82	0.009
RDM from café only	8	63	12.7	15	229	6.6	2.1	0.72–5.53	0.109
Cheese Ploughman's	0	63	0.0	8	229	3.5	0.0	0.00–1.71	0.133
Cake with fresh cream	3	63	4.8	23	229	10.0	0.5	0.08–1.56	0.192
Cheese sandwich	2	63	3.2	13	229	5.7	0.5	0.06–2.51	0.426
Scone with jam and fresh cream	6	63	9.5	29	229	12.7	0.7	0.24–1.90	0.497
Blue cheese with garden salad	1	63	1.6	3	229	1.3	1.2	0.02–15.4	0.867

a This variable includes all raw milk consumption, including milk added to coffee and tea while dining at the café.

Our multivariable logistic regression model included variables for raw drinking milk exposure, age and sex (Table 2).

**Table 2.** Multivariable analysis model for raw milk adjusted for age and sex

Exposure	Adjusted odds ratio	95% confidence interval	*P* value
RDM^a^	3.96	1.61–9.76	0.003
Sex	2.05	1.13–3.71	0.018
Age	0.98	0.96–0.99	0.005

a This variable includes all raw milk consumption, including milk added to coffee and tea while dining at the café.

### Microbiological results

There were 16 laboratory-confirmed cases of *C. jejuni* infection in this outbreak. Two RDM samples from the farm premises were positive for *C. jejuni*: one bottled sample (retail) from the self-service vending machine collected on 13 December 2016 and one from the RDM bulk tank collected on 22 December 2016. Not all 16 human isolates were viable for further testing; only seven human isolates were further typed by WGS.

Analysis of the seven-loci Multi Locus Sequence Type (MLST) derived from the WGS data showed that all of the seven viable *C. jejuni* isolates from human cases and both *C. jejuni* isolates recovered from RDM samples were all MLST CC ST-403 ST 7432; nine isolates in total. Furthermore, there were no SNP differences between all nine of these *C. jejuni* ST 7432 isolates indicating the strains were genetically indistinguishable. These results are consistent with a common source for the outbreak.

### Environmental results

During the outbreak investigation, inspections of Farm X by EHOs and DHIs took place on 13, 19, 22 and 29 December 2016, with further visits on 5, 10 January, 8, 15, 22 February and 1 March 2017. The dates of RDM sampling and legal enforcement prohibition requirements are shown in Figure 1.

During the visit on 19 December 2016 to Farm X, control methods were reviewed and the farm was requested to suspend RDM sales on a voluntary basis. A Hygiene Emergency Prohibition Notice was issued on 23 December to prevent sales of RDM; this was confirmed as a Hygiene Emergency Prohibition Order on 29 December 2016.

Environmental swabs taken from the milking parlour on 22 December 2016 and the water sample from the bore hole tested negative for pathogens. Nine clearance RDM samples were taken from the RDM bulk tank on 29 December 2016, they were negative for *Campylobacter* spp. but positive for Shiga toxin-producing *E. coli* O157 and *Listeria monocytogenes*.

A full assessment by DHIs on 10 January 2017 concluded that dairy hygiene practices were sub-standard at the premises and recommendations were made for Farm X to meet accepted hygiene standards. During a follow-up inspection to Farm X on 1 March 2017, improvements were observed by DHIs and dairy hygiene conditions were deemed to be satisfactory. Additionally, by March 2017, three sets of RDM clearance samples had been obtained, all of which were negative for pathogens.

## Discussion

This outbreak of *C. jejuni* included 69 cases and has been linked both epidemiologically and microbiologically to the consumption of raw cows' drinking milk produced and sold on a dairy farm in North West England. Investigations revealed that the raw milk contamination with campylobacter had likely begun in late October 2016 with the first few cases developing symptoms on 3 November 2016. We describe several issues that were identified during the outbreak investigation, including the inadequacy of current routine testing of RDM.

Over the last 5 years, the UK has seen a rise in the number of outbreaks of gastrointestinal illness linked to RDM consumption [14]. This has included several serious outbreaks with two involving *E. coli* O157; one during 2014 in Devon affecting nine people and the second during 2017 affecting seven people on the Isle of Wight [14, 22]. Concurrently, there has also been an increase in the number of RDM producers and volume of RDM consumed or sold in England. The most recent data from the FSA show that the number of registered RDM producers in the UK increased by over 50% during the past 4 years, from 108 producers in April 2014 to 168 in January 2018. There has been an even greater increase (400%) in the volume of RDM sold, from around 610 000 litres in 2012 to 3.2 million litres in 2017 [14, 23]. In addition to the upscaling of RDM production, farmers are also diversifying the ways in which they sell RDM to consumers; selling methods now include via the Internet [23] and via self-service vending machines [23] on their farm premises. Italy has seen outbreaks of campylobacter gastroenteritis associated with the use of raw milk vending machines [24]. FSA figures suggest in 2018 that there were 18 vending machines in operation in the UK [14]. The UK has seen a rapid expansion in RDM vending machine usage; during 2017, vending machines accounted for 17% of total RDM sales in the UK, compared to just 4% in 2012 [23].

A recent consumer survey commissioned by the FSA showed that there has been an increase in the prevalence of RDM consumption in the UK, with an estimated 10% of the UK population consuming RDM, compared to only 3% in 2012 [25]. This survey moreover found that nearly two-thirds of those consuming RDM do so either daily or weekly and that 11% of RDM consumers reported that a child or children under 18 years consume RDM products [25]. Costard *et al*. [26] assessed the disease burden associated with the consumption of RDM and reported that outbreaks associated with dairy consumption cause, on average, 760 illnesses per year and 22 hospitalisations per year. On reviewing data, they found that unpasteurised dairy produce caused 840 (95% confidence interval 611–1158) times more illnesses than pasteurised products. As this study assesses dairy consumption in the USA, it may not make it entirely generalisable to England; however, it indicates that with the increasing volume of RDM being consumed in England, we are likely to see an increase in outbreaks and cases of infection.

By law, sellers of RDM intended for direct human consumption must ensure their raw milk is routinely tested for coliforms and aerobic colony counts (ACCs) to comply with the conditions of their approval to sell [27]. The requirements under this legislation are a coliform count of <100 cfu/ml (colony forming units) and an ACC at 30 °C of ⩽20 000 cfu/ml. There are no specific legislative requirements for pathogen testing but Food Business Operators (FBOs) have a duty of care to ensure their food is safe under Regulation (European Commission) No 178/2002, so pathogen testing is recommended by the FSA. Despite this recommendation, pathogen testing is unlikely to be routinely carried out. If the shelf life is 5 days or longer, there is also a requirement to test for *L. monocytogenes* under Commission Regulation (European Commission) No 2073/2005 on the microbiological criteria for foodstuffs.

Under the terms of the approval granted by the FSA, coliform and ACC results must be seen by the DHI, who should also be informed of any failed results. Following unsatisfactory coliform or ACC results, the DHI, on behalf of the FSA, recommend the FBO cease sales until two follow-up samples (tested a week apart) give satisfactory results. This, however, is a recommendation only and is not enforced. Although these two microbiological parameters are useful for identifying indicator organisms, they are not indicative of all organisms that can contaminate the product during processing and samples that comply with the law and indicate good hygiene may actually contain pathogens [28, 29]. During this investigation, a bulk tank RDM sample complied with legislative standards, having ACC and coliform levels of 1060 and 15 cfu/ml respectively, despite also containing the pathogen *C. jejuni*. Consequently, the current testing regimes appear to be inadequate to find pathogens and are not a failsafe. Following this outbreak, a local survey was carried out in Lancashire by PHE FWE Laboratory, York over a 4-month period in 2017. Despite the low sample numbers (*n* = 59), the pathogens *Salmonella* Dublin and *C. jejuni*, and the Shiga toxin-producing *E. coli* O133:H4, which has an unknown pathogenicity, were all detected. Coliform and ACC results for these samples were satisfactory, providing further evidence that legally compliant samples are poor indicators of the presence of pathogens [30]. Willis *et al*. [28] describe the microbiological safety of RDM and found that nearly half of the samples tested contained either pathogens and/or indications of poor hygiene practice. The authors also tested successive samples from the same premises and demonstrated that the presence of pathogens or unsatisfactory levels of indicator organisms is not consistent over time, with pathogens detected from the same farm weeks apart, with negative results in between. Both these findings, and those from our investigation, support the need for regular microbiological monitoring to detect transient contamination with pathogens. Furthermore, we recommend reviewing the legal testing criteria to include pathogen assessment, to ensure campylobacter outbreaks such as this are prevented in the future.

Unlike other more commonly isolated *C. jejuni* CCs from cases of human infection such as ST-21, the ST-403 CC identified in this outbreak has only been reportedly isolated from cattle and pigs rather than from avian host species, the primary reservoir for *C. jejuni* [31]. This finding, together with the fact that WGS confirmed that the *C. jejuni* isolates from the RDM were identical in SNP analysis to the isolates from cases, provides additional evidence that RDM was the source of infection.

There is a legal requirement to include public health messaging with RDM to inform the public of the health risks. At the time of our outbreak investigation, the Food Labelling Regulations 1996 [32] required that RDM sold in England and Northern Ireland to have its container labelled with the following warning ‘This milk has not been heat-treated and may therefore contain organisms harmful to health’. The wording required in Wales is more detailed, explicitly underscoring the risk to vulnerable groups ‘The Food Standards Agency strongly advises that it should not be consumed by children, pregnant women, older people or those who are unwell or have chronic illness’ [33]. During our investigation, the lack of specific messaging for vulnerable groups was of concern and we are pleased to note that the FSA intend to bring in further regulations for England and Northern Ireland requiring new compulsory labelling that mirrors the health warning already in place in Wales [34].

During this outbreak, it became clear that RDM was sold from Farm X through numerous routes; in the farm café, from a vending machine, in the farm shop and at local farmers' markets. It was not clear through our investigation whether all farm café diners had the opportunity to read the RDM advice warning (as per the legal wording) before they ordered and consumed RDM, as a solitary sign with the advice wording was located at the café's till. The café was run using waitress service, with diners only visiting the till at the end of their visit. Incident recommendations included that warnings be printed on menus so that all diners had the opportunity to read the RDM advice warning.

In this outbreak, there was a substantial lag (nearly 6 weeks) between the onset of the two earliest confirmed cases and detection of the outbreak. This resulted largely from the way exposure data on campylobacter cases are collected. First, completion of the surveillance questionnaires is via post, which inherently creates a delay. Second, questionnaires are self-completed by cases and there is no follow-up of non-responders, so completion rate and quality of response are variable. Finally, since the surveillance questionnaire did not include specific questions about raw milk consumption, the earliest cases did not initially report drinking raw milk from Farm X; this was only identified when EHOs contacted them again following the identified link between two subsequent cases 2 weeks later. This time delay could potentially be shortened by using an electronic method to notify EHOs of cases, using an electronic questionnaire and including a question specifically on raw milk consumption.

## Limitations

Dissemination of the questionnaire for the analytical study via a pro-active press release was extremely efficient and quick: the story was picked up extensively in the local news and we received nearly 200 responses within 30 h of the release going out. One potential drawback with this approach, however, was that the widespread media coverage could have led to misclassification bias. First, people may have exaggerated their symptoms or exposures. Second, as there is a growing raw food movement [35] who believe strongly in the added benefits of uncooked, unprocessed food and drink, it is possible that some people completed the survey inaccurately to try to downplay the link between RDM and causing infection.

To simplify the questionnaire, we asked for details about visits to the café on or after 24 October 2016 but did not ask respondents to specify the date. We can therefore not be sure that for cases, consumption of RDM occurred in the 10 days before illness onset. However, there were only eight cases who only consumed RDM from the café; the remaining cases who had been exposed to RDM had also (or only) consumed it from the vending machine and we collected dates of purchase for exposure which was used as a proxy for date of consumption. We did not include dose–response questions during this outbreak as we wanted to keep the questionnaire as short and simple as possible. While this would have added further support for the hypothesis, we felt that as we already had microbiological results showing campylobacter isolated from the milk samples, this provided even stronger supporting evidence, making dose–response less relevant.

## Conclusion

Outbreaks of campylobacter have been associated with the consumption of RDM. This was a large outbreak which affected 69 people over an 11-week period, indicating a high level of raw milk contamination with campylobacter. The required warning label should include additional wording to emphasise the risk to vulnerable groups. The rise in RDM consumption, coupled with the growth of vending machines selling RDM in England, have increased the risk to the wider population by making unpasteurised milk more readily accessible. We cannot guarantee product safety with RDM; current testing regimes are not fit for purpose and the public remain at high risk when consuming RDM. The evidence generated from this outbreak should be used to influence and effect policy change.
